# Network-Based Delivery and Sustainment of Evidence-Based Prevention in Community-Clinical Partnerships Addressing Health Equity: A Qualitative Exploration

**DOI:** 10.3389/fpubh.2020.00213

**Published:** 2020-06-26

**Authors:** Shoba Ramanadhan, James Daly, Rebekka M. Lee, Gina R. Kruse, Charles Deutsch

**Affiliations:** ^1^Department of Social and Behavioral Sciences, Harvard T. H. Chan School of Public Health, Boston, MA, United States; ^2^Division of General Internal Medicine, Massachusetts General Hospital, Boston, MA, United States

**Keywords:** community-clinical partnerships, social networks, evidence-based interventions, implementation, equity, preventive services

## Abstract

**Background:** Increased delivery of evidence-based preventive services can improve population health and increase health equity. Community-clinical partnerships offer particular promise, but delivery and sustainment of preventive services through these systems face several challenges related to service integration and collaboration. We used a social network analysis perspective to explore (a) the range of contributions made by community-clinical partnership network members to support the delivery of evidence-based preventive services and (b) important influences on the ability of these partnerships to sustain service delivery.

**Methods:** Data come from an implementation evaluation of the Prevention and Wellness Trust Fund initiative, which supported nine Massachusetts communities to coordinate delivery of evidence-based prevention and address inequities in hypertension, pediatric asthma, falls among older adults, or tobacco use. In 2016, we conducted semi-structured interviews with (a) leadership teams representing nine community-level partnerships and (b) practitioners from four high-implementation partnerships (*n* = 23). We managed data using NVivo11 and utilized a framework analysis approach.

**Results:** Key network contributions for delivery of evidence-based preventive services included creating referrals, delivering services, providing links to community members, and administration and leadership. Less emphasized contributions included wraparound services, technical assistance, and venue provision. Implementers from high-implementation partnerships also highlighted contributions such as program adaptation, creating buy-in, and sharing information to improve service delivery. Expected drivers of program sustainability included the ability to develop a business case, ongoing network facilitation, technology support, continued integrated action, and sufficient staffing to maintain programming.

**Conclusion:** The study highlights the need to take a long-term, infrastructure-focused approach when designing community-clinical partnerships. Strategic partnership composition, including identifying sources of necessary network contributions, in conjunction with efforts from the outset to link systems, align effort, and build a long-term funding structure can support the required coordinated action around preventive services needed to improve health equity.

## Introduction

Clinical preventive services can reduce morbidity, early mortality, and costs to the healthcare system, but Americans typically receive only about half of the recommended preventive services (1, 2). Coordinated action linking clinical services, community offerings, policy action, and stakeholder engagement is required to address the multi-level factors that drive utilization of preventive services (3, 4). Community-clinical partnerships support such coordination and are prime channels for the delivery of prevention-focused evidence-based programs (EBPs). By EBPs, we mean programs, practices, and policies proven effective through rigorous research (5). Coordinated delivery of preventive EBPs through community-clinical linkages have impacted a wide range of behaviors, from diet and physical activity to tobacco and alcohol use (6). Community-clinical partnerships can have particular power for marginalized communities, leveraging strong relationships held by community-based organizations to deliver services to groups whose needs are not met by traditional public health and healthcare channels (7–9). These partnerships can also address two limitations of the current evidence base related to health equity: (a) limited reach or relevance of EBPs to marginalized communities and (b) insufficient attention to the context in which EBPs are delivered (10, 11). These challenges prompt an explicit focus on social determinants of health—the social, economic, and political forces that impact health directly and indirectly through the places where people live, learn, work, and age (12, 13). Thus, it is in the context of tremendous, but insufficiently tapped, potential that we consider community-clinical partnerships to deliver preventive EBPs and address health equity.

Coordinated action through community-clinical partnerships can marshal complementary human and social capital, improve information flow, reduce redundancies, and accelerate the adoption and implementation of EBPs (14–18). Strengthening linkages within existing systems, or creating new delivery systems as needed allows for resource exchange to address identified prevention needs, improve population health outcomes, lower future medical costs, and enable partners to spread implementation costs across multiple actors (17, 19–21). Accordingly, multi-sector integration of community-based preventive services is considered a key method to drive prevention (22), and accountable care organizations (ACOs) and other integrated care models increasingly incentivize coordinated preventive care delivery to reduce costs and improve preventive care outcomes (23). However, the impact of community-clinical partnerships is often reduced by community or clinical providers being unaware of the other groups' services due to a lack of pre-existing relationships, limited insurance coverage for integrated services, and insufficient infrastructure (e.g., referral systems) to connect workflow and processes between organizations (24).

A social network perspective offers insight into mapping community-clinical partnerships as social systems and finding intervention points to strengthen them and increase their impact. This lens prompts consideration of the benefits of inter-organizational networks, such as sharing risk, fostering innovation, and supporting responsiveness and flexibility. It also requires attention to barriers, which include difficulties in achieving consensus around goals, differences in culture, the significant investments required for relationship-building and network maintenance, and imbalances in power among partners (25). The network lens also prompts consideration of social capital, or resources embedded in social systems that support action (26). Systems that emphasize the flow of resources and information across sectors have become the norm for delivering social services and support the integration of programs into communities, coordination of services, ability to secure resources, and engagement with government actors (27–29).

To identify opportunities to build successful partnerships, it is critical to identify the set of resources exchanged that support the implementation of preventive EBPs. Network-based communication and influence, resource exchange, and engagement of critical stakeholders are important determinants of EBP implementation (30–32). A recent examination of coordinated service delivery through local partnerships highlighted a series of network-based contributions and resources: data, health and other expertise, connections to communities of interest, leadership, and policy influence (33). In addition to achieving short-term outcomes, partnership networks are vital for sustained service delivery in community settings (34). Long-term benefit to the community comes from program sustainability, or the extent to which partner organizations institutionalize selected preventive EBPs (35). Several characteristics impact sustainability of EBPs and the partnerships that support them, including redundancy of connections between organizations (to protect against turnover), presence of a “champion” in the network, participation from partners who can offer financial or administrative support, and the fit between the program and the network (15, 36, 37).

It is clear that much of the success of community-clinical partnerships depend on the network's ability to access, deploy, and sustain needed resources in an efficient manner. However, it is less clear what this means in terms of identifying the resources needed to support partnered service delivery and plan for program sustainability. Thus, we used qualitative inquiry to explore two questions to fill these gaps. First, what contributions by community-clinical partnership network partners support the delivery of preventive EBPs? Second, what do participants in a community-clinical partnership perceive to be the major influences on sustained delivery of preventive EBPs?

## Materials and Methods

Data for this study come from a mixed-methods implementation evaluation of the Prevention and Wellness Trust Fund (PWTF) initiative (38). This initiative was funded by the Massachusetts legislature through an assessment on payers and large hospital systems, and was directed by the Massachusetts Department of Public Health (MDPH). The MDPH released a call for proposals to create partnerships that addressed one or more of four of the most prevalent and costly priority conditions: hypertension, pediatric asthma, falls among older adults, and tobacco use. These conditions all had preventive EBPs available to address them that were not covered by insurance, and were determined by MDPH to have the potential to change health outcomes and reduce costs within 5 years. Partnerships were required to address the needs of at least one population group in their service area experiencing health inequities for a priority condition. Partnerships were also encouraged to center the role of community health workers (CHWs) to deliver services and help community members navigate healthcare systems, while reducing barriers to care driven by social determinants of health, such as housing, transportation, and discrimination. A total of 16 EBPs were available for selection. The full list is available elsewhere, but the list of interventions for pediatric asthma illustrates the diversity of options: care management (a multi-prong intervention), asthma self-management education in primary care, home-based care led by CHWs, and comprehensive asthma management offered in schools or via Head Start. MDPH provided technical assistance, learning group sessions, and quality improvement support to each partnership. By focusing on populations experiencing inequities, taking a multi-level and systems approach, and addressing social determinants of health, the PWTF initiative was designed to address health equity (38–40).

The primary evaluation focused on the reduction in preventable health conditions in the four target areas, reduction in associated healthcare costs, and identification of the communities that benefited from the services. As a complement, the mixed-methods implementation evaluation focused on barriers and facilitators of implementation, as well as implementation outcomes. We drew on the Consolidated Framework for Implementation Research (41) to guide the study. This framework highlights key determinants of barriers and facilitators of successful implementation, grouping these drivers into those related to the intervention, outer setting (e.g., broader context), individuals involved with implementation, inner setting (e.g., organizational context), and process of implementation. The framework highlights social networks in the context of the outer setting (e.g., relationships with other organizations) and the inner setting (e.g., the connections within the organization that support flow of information, power, and resources).

A total of nine partnerships across the state were chosen to deliver preventive EBPs, with support and technical assistance from the MDPH. Each partnership included at least one city/regional planning agency, one clinical health provider, and one community-based organization. The nine partnerships covered populations ranging in size from 40,000 to 140,000 people. Some partnerships covered single cities, others included multiple cities and towns, and one constituted an entire county. About 900,000 people, or 15% percent of the state's population, resided within the nine funded partnerships' service areas. Compared to state averages, the selected communities had higher prevalence rates of the priority conditions, representation of racial and ethnic minorities, and poverty rates (42). Additional details describing the initiative are provided elsewhere (38, 39).

The present study focuses on social networks held within each of the nine partnerships. The partnerships were composed of a coordinating partner (or lead organization) and several local organizations. The programmatic activities for PWTF started in January 2015. The evaluation components described here took place in the spring and summer of 2016, when the partnerships were one to one and a half years into the implementation effort. The Harvard University Institutional Review Board determined that full review was not required for this study. The study was also deemed exempt from review by the Massachusetts Department of Heath Institutional Review Board. We have utilized the COREQ guidelines for reporting qualitative research to ensure thorough, transparent description of our methods (43).

### Phase 1: Interviews With Coordinating Partners

Three members of the study team (SR, RL, and GK), who are trained qualitative researchers, conducted semi-structured interviews with the leadership teams of the nine coalitions in March 2016. We did not have established relationships with participants before the study. The phone-based interviews took about 90 min to complete. For each site, we requested that the individual leading the partnership and at least one person involved in day-to-day operations participate. For eight partnerships, the interviews were conducted with two individuals and for one partnership, four individuals participated. These in-depth discussions supported our exploratory research goals by offering rich insight into participants' perceptions and views (44).

We defined network boundaries for each partnership as the set of organizations involved with PWTF implementation (45). For each partnership, we started with the list of organizations provided by the MDPH and then reviewed it with coordinating partners, who made changes to the list as needed. We asked about each network member in terms of their offerings related to EBP implementation, referred to henceforth as network contributions. By network contributions, we mean resources offered to support the partnership, such as information, leadership, or connections to community members. We explored an initial set of network contributions based on the structure of the overall grant (e.g., provide referrals or deliver clinical services) and the theoretical framework. We also asked participants to share other network contributions. We asked questions about partnership formation processes and their expectations regarding partnership sustainability after the funding cycle ended. The interview guide is available as part of the study's methods protocol (38).

### Phase 2: Interviews With Implementers From High-Implementation Partnerships

After completing the interviews with coordinating partners, the team fielded an online quantitative survey that included self-reported data regarding the extent to which the selected EBPs had been implemented. We measured the degree of implementation using a 4-point Likert scale drawn from the work of Fernandez and colleagues (46) with the following response categories: (0: No implementation, 1: We are at the early stages of implementation, 2: We have implemented this strategy, but inconsistently, or 3: We have implemented this intervention fully and systematically). We aggregated scores for each EBP by averaging responses from all respondents in a given partnership. We then identified four partnerships that had scores for all EBPs within one priority condition that were higher than the partnership-wide average. Additional details about the quantitative assessment are provided elsewhere (38). Given resource constraints, we focused on the high-implementing partnerships, to identify resources exchanged and considerations for sustainment.

For each of the four high-implementation partnerships, we (SR, JD, RL) used a purposive sampling strategy and conducted semi-structured key informant interviews with four to six program implementers, including at least one clinical partner and one community partner. Interview guides focused on the network contributions of PWTF partners and assessment of community-clinical linkages, facilitators and barriers to implementation, and expectations around sustainability. The interviews took about 1.5 h to complete and were conducted in-person from July to August 2016. Given that we were interested in the opinions of staff from different organizations and with different levels of seniority, we chose to use key informant interviews (rather than focus groups) to ensure that a diversity of perspectives could be shared openly. All individuals invited to participate accepted the request. All interviews were audio-recorded and transcribed verbatim by a professional transcriptionist.

### Data Analysis

The analytic plan was grounded in the framework analysis approach (47, 48). The research team used a two-stage coding process that included both prefigured and emergent codes. Members of the study team (including JD and RL) conducted the initial coding, which was primarily descriptive, using the Consolidated Framework for Implementation Research as the framework for the prefigured coding structure. The second phase of coding used a more inductive approach, and the coding structure was refined by the broader study team (including SR, GK, and CD). These refined categories formed the broader thematic framework, which was then applied to all transcripts. After drafting initial results and interpretations, we shared these interpretations with members of the PWTF partnerships and refined our interpretations based on their feedback. For this analysis, additional coding was conducted by JD and SR to focus on network contributions and drivers of sustainability. We utilized NVivo 11 to organize and retrieve data (49). Our team brought a range of perspectives to the analysis. All members are actively engaged in public health research, focusing on implementation science and emphasizing community-engaged research. With this history, the team had an appreciation for the complexity of integrating EBPs into practice settings and the disconnects between theory and practice related to the adoption and implementation of evidence-based preventive services.

## Results

The partnerships ranged in size from five to 15 participating organizations, with an average of 10 organizations per partnership (standard deviation =3.41). Of the four core health topics, partnerships addressed between two and four, with an average of three. For some partnerships, all members were active on all selected health topics, but for other partnerships, different subsets of members addressed different health conditions. As seen in Table 1, the nine partnerships included a wide range of types of organizations.

**Table 1:** Representation of different types of organizations across the nine partnerships (*n* = 91 organizations).

**Organization Type**	**Total N (%)**	**Mean number per partnership (range)**
Clinical	33 (36.3%)	3.7 (1–7)
Community/Schools	31 (34.1%)	3.4 (1–5)
Government/Planning	21 (23.1%)	2.3 (1–5)
Other^*^	3 (3.3%)	0.3 (0–2)
Unclassified	3 (3.3%)	0.3 (0–2)
All Organizations	91 (100%)	10.1 (5–15)

* *Other organization types included research centers at academic institutions and a community senior center*.

In Phase 1, nine “coordinating partner” interviews were conducted with twenty participants representing a range of roles, including project managers and directors, chief executive officers or vice presidents of health centers and systems, and directors of local health agencies. Each interview included one person who was responsible for the leadership of the partnership. There was also a wide distribution of roles among the 23 staff implementers interviewed in Phase 2. Many of these roles were client-facing, such as CHWs, program coordinators, physicians, nurses, tobacco treatment counselors, community navigators, and an attorney. Other participants interviewed held more administrative roles, including a school district nursing director, associate and executive directors at community-based organizations, a CHW coordinator, and a health center practice administrator.

**Figure 1:**
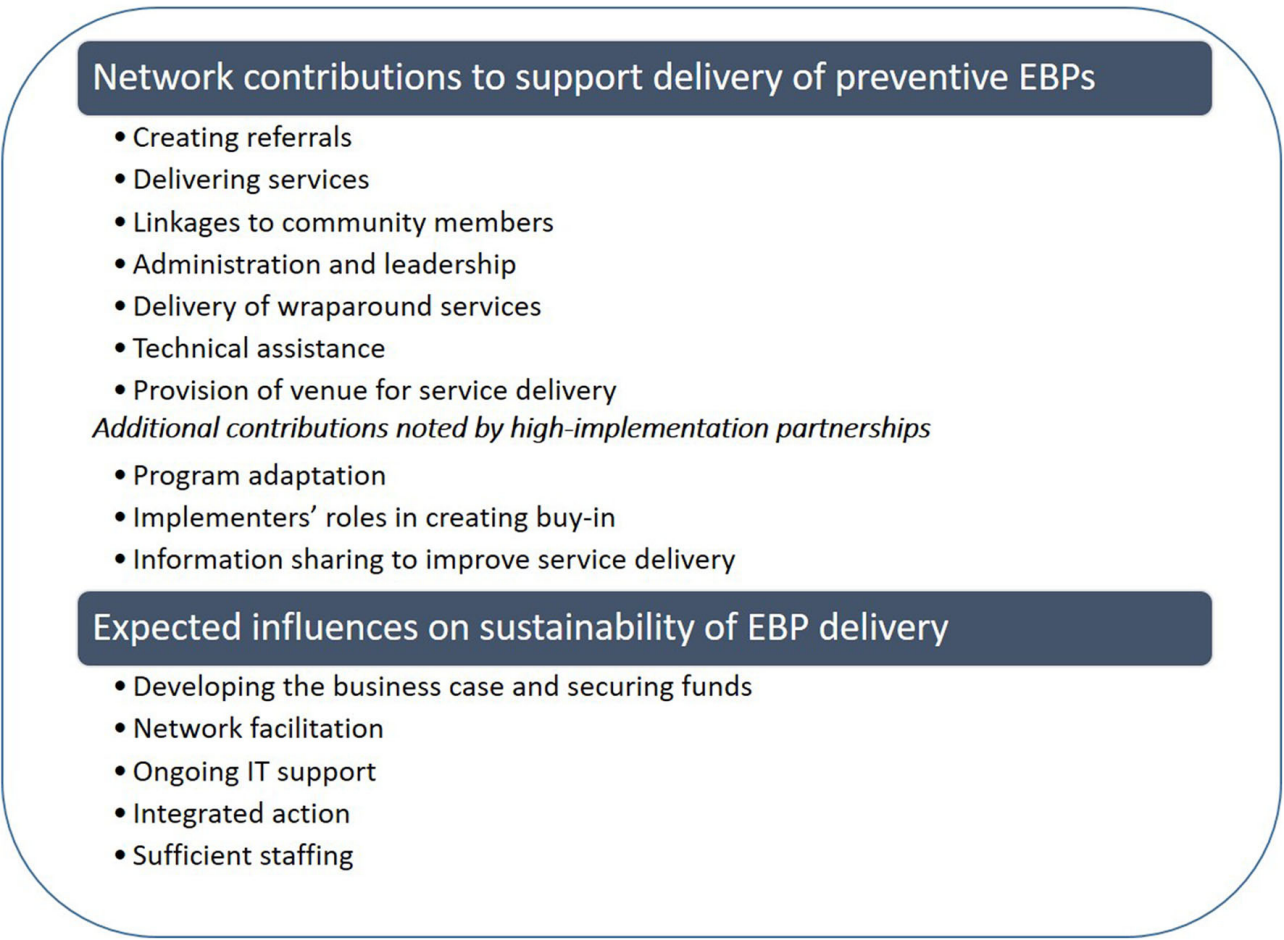
Key themes identified in the analysis, organized by topic and presented in order of decreasing emphasis.

As seen in Figure 1, we present findings in two main areas: (1) network contributions that support preventive EBP delivery and (2) key influences on EBP sustainability.

### Key Network Contributions to Support Delivery of Preventive EBPs

Participants highlighted several key resources offered by partners to support EBP delivery through community-clinical partnerships, presented here in decreasing order.

#### Referrals

Referrals were the most frequently discussed network contribution, which was fitting given their centrality to the community-clinical partnership model utilized by the PWTF. Participants described using both electronic referrals as well as paper forms sent by fax or courier. E-referrals were meant to be the main method of sharing referrals, but difficulties in connecting systems between organizations (due to technical issues and privacy limits) were a hindrance for several participants. Participants noted that a large amount of time/resources was required to modify electronic health records (EHRs) to support referrals. Referrals for the PWTF effort included referrals from clinical to community settings, community to clinical settings, and “reverse referrals,” when a community organization would identify a health need for a client and request a referral from the client's provider to deliver services. Several participants noted that successes related to broadening the range of staff members or partners who created referrals. A few participants mentioned that organization of the referrals by the coordinating partner also allowed them to take advantage of referrals more easily.

#### Clinical or Programmatic Services

Delivery of behavioral and clinical services was central to the PWTF model and was an important network contribution identified by most participants. Depending on the EBP and priority condition, these services ranged from assessments and screenings to smoking cessation counseling and asthma action plans.

#### Linkages to Community Members

Participants described the tremendous amount of effort needed to establish a presence in the community as a trusted source. Participants emphasized the high volume of outreach activities, attendance at community events, connections with CHWs, connections with organizations, etc. needed to maintain a presence and effectively conduct outreach. They also highlighted the benefit of having consistent public-facing staff, e.g., outreach coordinators or CHWs, serve multiple programs so that investments accrued across health issues.

“*One of the pieces that we're trying to do is really get CHWs not just here at [organization] but like across the whole partnership into the community more, so at community events, at community fairs, setting up outside like [organization], which is our grocery store sort of down in our low-income neighborhood and going to housing authority that kind of stuff to really let people know about the work, really work to refer and engage them back into one of the programs. So that's really been our biggest effort around health equity is really the engagement of our CHWs out into the community.” (Implementer, 4)*

Finally, many participants noted the importance of leveraging local connections, such as with community organizations dedicated to serving a particular population to broaden their reach. A few participants noted that confidentiality issues impeded their ability to work across organizations. As an example, one implementer described a close relationship with a tobacco treatment counselor who was part of the partnership. However, if a client engaged with the implementer / organization around quitting tobacco, it was not possible to follow up with the tobacco treatment counselor about the client's status without having anticipated this at the outset and obtained consent.

#### Administration and Leadership

Participants noted the importance of having sufficient leadership to move the range of partnership components forward in a cohesive manner. Key aspects of administration and leadership included the ability to create buy-in, embrace a diversity of perspectives, and engage high-level leaders from participating organizations. A number of participants noted that the structure of the partnership, which included multiple touchpoints and opportunities to connect, supported their ability to provide coordinated services.

“*The medical center provides for our partners …centralized coordination for QI, so our quality improvement for our community-based organizations and our clinical teams all fall under the [Name] coordinating partner role” (Coordinating partner, 7)*.

#### Delivery of Wraparound Services

A smaller number of participants noted the wide range of services offered by partners that supported the community-clinical partnership to address barriers in access to care that were related to social determinants of health. Action in this area included connections to health insurance, navigating the healthcare system, connecting clients with childcare, housing, food access, additional resources (e.g., extra bedding to reduce laundry burden for asthmatic children), environmental assessments, pest control, financial management, and legal action against landlords violating renters' rights.

“*We've actually had some great successes in helping families retrieve back rent, finding suitable housing facilities. Also, they've helped them with–helping with moving, helping to break a lease when the environment is not conducive to the health of the child who has asthma.” (Coordinating partner, 9)*.

#### Technical Assistance

Responses related to technical assistance emphasized the range of contributions offered by MDPH and other partners to fill a given organization's gaps. These supports included insight on legal issues, quality improvement, racial justice, program implementation, housing sector information, sustainability, and data mapping. Participants emphasized the ability to fill in gaps of expertise or resources and general support for their efforts.

“*They're there constantly checking in on us to make sure that we have all the resources, all the support that we need from our organization and kind of like just there to help us be successful.”(Implementer, 4)*.

#### Venue

Participants noted that some partners provided a venue, such as space to hold a class or community event. This was not emphasized as an important contribution.

##### Network contributions identified by implementers from high-implementation partnerships

In addition to the themes described above, implementers from high-implementation partnerships also emphasized a unique set (ordered in decreasing emphasis): program adaptation, creating buy-in, and information sharing to improve service delivery.

#### Program Adaptation

Many participants discussed the need to adapt EBPs to meet the needs of the populations they serve, with three types of adaptations. First, a common type of adaptation related to making changes to program materials to better meet the needs of populations experiencing health inequities. Exemplar changes included translating materials into other languages and simplifying language/using visuals to meet the needs of a broader audience. Second, a series of adaptations were made in terms of the dose or format of the intervention, again with a goal of increasing acceptability of the EBP to the population of interest. For example, participants noted the need to make interventions shorter or less intense or pairing the EBPs with other health topics of greater interest to the population as a means to start the conversation.

“*Well we added the wellness fairs to deal with the smoke-free housing. And those wellness fairs, instead of just being focused only on tobacco treatment we did present the other interventions and included a couple other local agencies we thought would pull people out of their apartments…You are not telling me to quit smoking. Oh but you have a wellness check? I can check my cholesterol and my blood sugar and my blood pressure? …It's sort of like gentle and not holistic but inclusive.” (Implementer, 7)*.

A third set of adaptations were made to support delivery through a new system/community-clinical partnership model. Many of the adaptations made were linked to program logistics, scheduling, and administrative processes. Some of these adaptations were required to move the program from a community- or clinically-based program into a community-clinical partnership delivery model.

“*So when I created the workflow for [the EBP], I did a lot of research through the [shared electronic drive]. I got a lot of CDC materials on them. They offered me I guess the base of what the [EBP name] screening was. …Through research, I tried to figure out what was the best way to create this workflow and create something that works well with the medical system… There was no way of knowing how to integrate the community interventions with the clinical side because it just hasn't been done. But it's just taking similar models and remodeling them.” (Implementer, 2)*.

#### Implementers' Efforts to Secure Buy-In Among Collaborators

Many implementers highlighted their efforts (and related challenges) in the area of creating buy-in to support EBP implementation. As a complement to efforts by partnership leaders to increase buy-in, implementers noted that they were often in a position of needing to introduce the program to clinical collaborators and convince them to take part, e.g., at staff meetings or through site visits. Implementers' efforts in this regard centered on (1) increasing buy-in for the role of CHWs or other non-physician staff and (2) garnering support among the clinical partners for delivery of EBPs through community agencies. Participants noted challenges of introducing a model that relies on CHWs and other non-physician staff and actively promoting integration into the larger system. For systems that did not have CHWs previously, there was a need to generate support for the role, as noted below:

“*So there was a lot of education too around what is a community health worker… How is that going to benefit me as a provider?.Until they actually saw that it actually worked they didn't really process it the same way… I think [the challenge] was more buy-in to the CHW role…we already had smoking cessation. They didn't have to buy into that.” (Implementer, 7)*.

Other participants described challenges in clinical settings because of insufficient value placed on the role of the community sector and EBPs delivered by community-based organizations (CBOs). This was described as inhibiting the development of the partnership model and was thought to result in fewer referrals than intended. Participants also highlighted the challenge of bringing implementing partners on board with the new EBP:

“*The biggest miss and frustration has been that we have this grand idea of the doctors are just going to love it, and they'll refer people to these community programs, and it will just be wonderful. In reality that is a long way off….The lack of the clinical buy-in has just been the biggest piece of it that's hard because it's on the community end we have to work a lot harder to get those relationships.” (Implementer, 2)*.

#### Information Sharing to Improve Service Delivery

Participants highlighted the sharing of information to support better service delivery as an important network contribution. One type of information shared related to successes and new approaches, so that other members of the partnership could benefit from these learnings. In this regard, participants highlighted opportunities to connect with partners with greater expertise in a health topic, e.g., tobacco, or with a particular population. Another type of information shared was up-to-date clinical and service information. Finally, participants noted that sharing information (separate from referrals) offered opportunities to track clients beyond a small set of encounters. This was expected to have an impact on organizations' ability to see the impact of new investments or track a given client's progress through the system.

“*The hub actually reached out to the patients, set them up for the appointments, took that out of the hands of my staff having to then track them down, then relayed back the information to the providers. So then we also knew–did they go to the appointment? Did they not show up for the appointment? They went to the appointment, and this is what happened…I think the hub played a very big part in helping us to get our patients to where they needed to go. (Implementer, 7)*.

One participant noted that confidentiality and privacy protections limited the sharing of data (in her example, from schools to clinics) that would have otherwise supported a more holistic process for managing client needs. Several participants highlighted the many different systems they were trying to work with to improve communication, but electronic health record (EHR) systems still proved to be a major barrier that inhibited the sharing of data both across and sometimes within organizations.

“*–and the EHR interoperability with hospital systems. So the fact that we can't communicate well… The whole point of this is to reduce ED visits. We have to have the ED visit data, and we can't figure out a good way to get that data.” (Implementer, 9)*.

### Sustainability of Preventive EBPs Delivered Through Community-Clinical Partnerships

When asked about the potential sustainability of PWTF programs and services after the funding period ended, participants highlighted a number of likely influences (presented in order of decreasing emphasis): developing the business case and securing funds, network facilitation, ongoing IT support, integrated action, and sufficient staffing.

#### Developing the Business Case and Securing Funds

Concern about securing future funding to support these clinical-community partnerships and implementation of EBPs was the most commonly cited barrier to sustainability among participants. Reactions were mixed regarding the potential to sustain the specific EBPs, with many participants noting in equal measure a desire on the part of partners to continue offering the service and a challenge once the funding stream ended. Many participants indicated that key aspects of the partnerships would be discontinued or scaled back if they were unable to secure alternative funding sources. CHWs and clinical-community referrals, particularly to non-billable EBPs, were the more frequently mentioned activities of the partnership at risk.

“*It's an important question …how much of this can be paid for through reimbursement from insurance companies versus programs like this. I mean, you know, the amount of time I think that the community health workers–they put into going to homes and doing these home visits I'm sure is pretty huge. And I don't know how reimbursable some of that stuff is. And that to me is a huge piece of what's going on.” (Implementer, 9)*.

Strategies for developing the business case and securing funds mainly centered around demonstrating to payers (e.g., private insurers, Medicaid, and Medicare, state agencies) the benefits of these prevention programs and potential for healthcare cost savings and leveraging healthcare's shifting landscape and government incentives or mandates associated with ACOs and other integrated care models. Several participants at clinical organizations described how these integrated care models might allow billing codes to be applied more broadly to support sustaining the delivery of certain EBPs.

“*A lot of those office visits and those sort of nonclinical wellness checks through some of the programming that we have now or that's coming down the pike sort of –the reimbursement structure is changing for healthcare –and our primary care sites are becoming Patient-Centered Medical Home, so it sort of allows for those visits, so some of those visits will be sustained. There's also some chronic disease billing codes that we're looking into to work with sustainability. We've found the role of the CHW to be invaluable. And so we'll sort of like find a way to get them either reimbursed or funded through other sources.” (Implementer, 4)*.

#### Network Facilitation

Participants highlighted the need for a coordinating body to maintain the high level of collaboration employed by these partnerships, particularly between clinical and community sites, suggesting that without this support, certain processes were unlikely to be sustainable. Many participants noted that although there would be a desire to continue the work and an intention to continue coordinating action, it would be unlikely to happen without a designated leader. One partnership strategically set up a “hub” within the health system to facilitate clinical-community linkages by supporting the referral process and patient follow-up before and after visits.

“*In our market, many of our practices are smaller in size… so it's very hard for our practices on the clinical side to develop the capital necessary to have embedded community health workers or to support–fully support some of the needs in terms of IT and quality. So we–part of the hub and part of this infrastructure was purposeful in creating a more centralized infrastructure that would leverage those necessary resources, navigation coordination and connection with the community in ways that we knew they would not be able to do on their own.” (Coordinating partner, 7)*.

#### Ongoing IT Support

Participants indicated that ongoing IT support would be essential to sustaining EBP delivery through these partnerships by facilitating collecting data, sharing information, and creating or executing bi-directional referrals between clinical and community organizations. While some participants reported e-referrals not functioning well, most, including those using e-referrals, expressed a need for continuous quality improvement to increase ease of use. Several participants discussed a need for support to set up e-referral systems (particularly the bi-directional functionality) and develop easy-to-use communication channels for organizations to access and share information.

Others mentioned the need to leverage technology and electronic systems to encourage and facilitate bi-directional referrals and optimize clinical workflows, including streamlining steps in the EHR. Participants in the partnership that created a hub system also expressed a need for additional IT support to facilitate system integration and centralized infrastructure. Also discussed was the role of care directors, who supported communication between clinical & community partners using EHRs.

“*We have what we call care directors … which allows our clinical and our community to communicate, and that piece alone I think is a real major change in the way that we do healthcare including our community-based organizations, and that's one of our places as we move the pendulum from disease-oriented to prevention, and prevention includes community partners and not just the hospital. So I think that's a huge place for us to be and certainly in the future.” (Coordinating partner, 7)*.

#### Integrated Action

Participants highlighted the connections built among organizations in the partnerships, many of which had not collaborated at all in the past or to such an extent. Newly developed community-clinical linkages and communication channels were viewed as an integral part of sustaining EBP delivery. A coordinating partner described the ways in which original boundaries between organizations were diminished through the coordinated action around EBPs.

“*So you have these relationships with your [elder agency] and your CBOs, with your hospital partners…And there's a certain lane that they're all in … like an Olympic size swimming pool, and the tethers and the buoys are in between the lanes…And if you thought about life before… PWTF and life after PWTF essentially what it is, is you're taking away the buoys and the demarcation in between the lanes…” (Coordinating partner, 2)*.

#### Sufficient Staffing

Participants perceived maintaining sufficient staffing for multiple roles as critical to sustaining partnership collaboration and successful EBP delivery within a partnership. The most frequent among the roles discussed pertained to the CHW-led community outreach, which precipitated a need for additional staff support coordinating patient referrals and delivering EBPs. Another challenge related to finding and retaining staff for EBP delivery discussed was the negative impact of staff turnover, which often left existing staff overburdened while training new staff. This was largely discussed regarding CHWs, a position of perceived high importance within both clinical and non-clinical settings, but which had high turnover and was generally perceived as understaffed. One explanation for this was that CHW services are typically not billable.

“*I can't believe how few CHWs we have, not just for the intervention, just in general. I mean that's by far the most important role in our clinic, any community health center in any health clinic. You need people that are community health workers. They're invaluable. So it seems hard to get buy in in that sense even from our like high, high up people. We've kind of met a wall I guess in trying to get more CHWs hired. And why is that? I think the response we get back is that it's not a billable visit.” (Implementer, 9)*.

## Discussion

This study examined community-clinical partnerships delivering preventive EBPs to explore the range of network contributions that supported EBP implementation and identify potential drivers of sustainability. A wide range of network contributions supported EBP delivery in the partnerships. Many of the contributions identified by the full group (e.g., creating referrals, offering connections to community members, and providing technical assistance) were consistent with the literature relating to community coalitions and partnerships for prevention (4, 50) as well as the underlying theoretical framework (41). However, the additional network contributions identified by the implementers offer important insight into considerations that may be unique for EBP implementation and community-clinical partnerships. For example, the need to adapt EBPs for delivery through a community-clinical partnership model is unique and the resulting changes to workflows and administrative processes may require customized supports. For this initiative, the pool of EBPs from which grantees could select did not necessarily include a role for CHWs, thus this was a point of potential adaptation for partnerships. Supports for adaptation will allow implementing organizations to navigate a balance between fidelity to the EBP as originally described and the necessary adaptations to increase fit and relevance for the new delivery model (51). That idea of balance also applies to the adaptations made to programmatic materials and exposure levels, framed here in the context of making the EBP more appropriate for populations experiencing inequities.

Another unique network contribution identified in this study was the set of activities by non-clinician implementers to create buy-in for the program among clinical staff, which offers an important point for further inquiry. The perception that for some partnerships, clinical partners did not place sufficient value on services delivered by non-physician staff (including CHWs) or through community-based organizations is a challenge to a referral-based program and more broadly to a sense of trust, reciprocity, and joint action within a partnership. Service delivery by CHWs—particularly in the context of care delivery by multidisciplinary teams—provides an important opportunity to improve health equity and reduce healthcare costs to payers (52, 53). The network contributions identified in this study can serve as a useful starting point for defining relationships in quantitative social network analyses to describe the functioning and impact of community-clinical partnerships delivering preventive EBPs.

As an extension of program implementation, the sustained delivery of preventive EBPs after initial funding expires presents a critical challenge. While the literature on this topic is still emerging, our findings were generally consistent with existing research regarding the key factors that influence the sustainability of EBPs in community and healthcare settings (54–57). As expected, the ability to create a business case and secure funds was a top priority, particularly in terms of funding roles and services that cut across organizations, such as the work of the CHWs. The results suggest a need for experimentation with a variety of payment models and payers (e.g., government, insurers, ACOs, foundations, etc.) to support both sustained EBP delivery and infrastructure for clinical-community partnerships beyond the short-term grant funding typical in public health (21, 58, 59). The emphases on network facilitation and ability to integrate services were also in line with the literature on coordinated community action, which emphasizes the importance of a coordinating actor and suggests that this role is best played by a trusted local entity, ideally with a geographic presence that overlaps with the partners (19, 21). Finally, the focus on data and IT services has been highlighted elsewhere as key to partnerships' ability to share information, collaborate, innovate, and respond effectively to constituents' needs (19, 60). A recent analysis of collaborative community networks found that technology and fiscal management/funding were the least commonly shared resources, suggesting an important opportunity for intervention (61).

Taken as a whole, study results point to the need to take a long-term, infrastructure-focused approach. After all, we must consider the moving parts that influence EBP implementation, including those highlighted by the theoretical model (41) as well as the ways in which those parts interrelate and form something that is larger than the sum of its parts (62). This infrastructure focus inherently emphasizes the strategic creation and management of community-clinical partnership networks, requiring a balance between number and diversity of members and the price of maintaining and engaging the network. Effective execution of the partnership requires attention to ensuring alignment of partner interests, creating consensus, ensuring partners achieve relevant benefits, and managing conflicts and disconnects (63, 64). As highlighted by a recent evaluation of multi-sector partnerships, it is a challenge to create efficient organizational structures to allow organizations to coordinate activities over the long-term and ensure a balance to upstream and downstream factors (65). At the same time, the infrastructure focus also addresses an ongoing need to build capacity in local systems, so that partnerships are able to address current and future health needs with evidence-based services (34). As part of this work, it may be important to build capacity around the selection of implementation strategies, or the set of actions that support EBP integration into organizations (5). There is a growing body of work describing the strategic selection of implementation strategies and this can be customized for community-clinical partnerships focused on prevention (66, 67). Building a system also allows for the network of partners to innovate and adapt, which can have important impacts on addressing the needs of marginalized populations (60, 68). Finally, a long-term perspective that focuses on the system is needed to ensure that funding for service delivery and partnership maintenance is protected. This is not the norm for multi-sector partnerships at this time, but could be an important area of inquiry given the impact on sustainable service delivery (65).

As with any study, there are a series of limitations. First, due to resource constraints and the need to limit respondent burden, we were only able to conduct implementer interviews within four high-implementation partnerships, rather than the full set of nine partnerships. Second, we conducted the evaluation when partnerships were roughly halfway through the implementation period and thus our identification of high-implementation partnerships does not reflect ultimate implementation levels. However, from a sustainability planning standpoint, this seemed to be the appropriate point at which partnerships should plan for the future. Third, we did not attempt to connect implementation levels with health outcomes and thus are unable to comment on the broader impacts of EBP delivery. The limitations are outweighed by the strengths of the study. First, the study focuses exclusively on partnerships working with marginalized communities and offers insight into partnership networks designed to increase health equity. Implementation science studies focused explicitly on marginalized communities will be critical to achieving health equity, but are limited at this time, thus the study adds to an important body of work (10). Second, participants had a wide range of roles in the PWTF initiative and thus offered complementary perspectives from implementers to leadership. Third, the nine partnership networks under study encompassed a great deal of diversity and therefore allowed for a broad range of network contributions to be uncovered. Finally, the network-level perspective provides an important addition to the literature on community-clinical partnerships, which typically focuses on short-term outcomes, rather than organizational linkages, or community infrastructure (69).

In summary, the study suggests a need for a long-term, systems-oriented view of network and infrastructure development in community-clinical partnerships to support the delivery of preventive EBPs. Long-term investments, centered around leadership and infrastructure maintenance, as well as a viable funding strategy, would allow for coordinated action around preventive services to improve health equity.

## Data Availability Statement

The datasets generated for this study will not be made publicly available as the data in our qualitative database can be traced back to individual subjects, which could put their privacy at risk.

## Ethics Statement

The Harvard University Institutional Review Board determined that full review was not required for this study because although the data were recorded in a manner in which human subjects could be identified, the disclosure of such responses outside the research could not reasonably be considered to place participants at risk. The requirement for written informed consent from the participants of the study was also waived by IRB staff. The Massachusetts Department of Heath did not require additional Institutional Review Board review as it was considered program evaluation.

## Author Contributions

SR, JD, RL, GK, and CD conceptualized the study. SR, JD, RL, and GK designed the interview guides and collected the data. SR and JD analyzed the data and drafted the manuscript. RL, GK, and CD edited the manuscript. All authors have approved the final version and take responsibility for the content.

## Conflict of Interest

GK declares that a family member has a financial interest in a health technology company that supports community health worker workflows—Dimagi, Inc. The remaining authors declare that the research was conducted in the absence of any commercial or financial relationships that could be construed as a potential conflict of interest.
